# Neurofluids and the glymphatic system: anatomy, physiology, and imaging

**DOI:** 10.1259/bjr.20230016

**Published:** 2023-06-01

**Authors:** Danny JJ Wang, Jun Hua, Di Cao, Mai-Lan Ho

**Affiliations:** 1 Mark & Mary Stevens Neuroimaging and Informatics Institute, Keck School of Medicine, University of Southern California, Los Angeles, United States; 2 The Russell H. Morgan Department of Radiology and Radiological Sciences, Johns Hopkins University School of Medicine, Baltimore, Maryland, United States; 3 F.M. Kirby Research Center for Functional Brain Imaging, Kennedy Krieger Institute, Baltimore, Maryland, United States; 4 Department of Biomedical Engineering, Johns Hopkins University, Baltimore, Maryland, United States; 5 Nationwide Children’s Hospital and The Ohio State University, Columbus, Ohio, United States

## Abstract

First described in 2012, the glymphatic system is responsible for maintaining homeostasis within the central nervous system, including nutrient delivery, waste clearance, and consistency of the ionic microenvironment. It is comprised of glial cells and barrier systems that modulate neurofluid production, circulation, and exchange. Experimental interrogation of neurofluid dynamics is restricted to *ex vivo* and *in vitro* studies in animals and humans, therefore diagnostic imaging plays an important role in minimally invasive evaluation. This review article will synthesize current knowledge and theories regarding neurofluid circulation and implications for neuroimaging. First, we will discuss the anatomy of the neurogliovascular unit, including paravascular and perivascular pathways of fluid exchange. In addition, we will summarize the structure and function of barrier systems including the blood–brain, blood–cerebrospinal fluid, and brain–cerebrospinal fluid barriers. Next, we will mention physiologic factors that yield normal variations in neurofluid circulation, and how various disease pathologies can disrupt glymphatic drainage pathways. Lastly, we will cover the spectrum of diagnostic imaging and interventional techniques with relevance to glymphatic structure, flow, and function. We conclude by highlighting current barriers and future directions for translational imaging and applications to neurologic disorders.

## Introduction

First described in 2012, the glymphatic system is responsible for maintaining homeostasis within the central nervous system (CNS), including nutrient delivery, waste clearance, and consistency of the ionic microenvironment. It is comprised of glial cells and molecular barriers that modulate neurofluid production, circulation, and exchange. Research into these complex systems has rapidly increased in recent years: the search term “glymphatic” yields over 1200 publications on PubMed, 93% of which have been published since 2017.^1^ Experimental interrogation of neurofluid dynamics is restricted to *ex vivo* and *in vitro* studies in animals and humans, therefore diagnostic imaging plays an important role in minimally invasive evaluation. This review article will synthesize current knowledge and theories regarding neurofluid circulation and implications for neuroimaging. First, we will discuss brain compartments, pathways for neurofluid circulation and exchange, and anatomy of the neurogliovascular unit. In addition, we will summarize the structure and function of barrier systems including the blood–brain, cerebrospinal fluid, retina, labyrinth, spinal cord, and nerve barriers. Next, we will mention physiologic factors that yield normal variations in neurofluid circulation, and how various disease pathologies can disrupt glymphatic drainage pathways. Lastly, we will cover the spectrum of diagnostic imaging and interventional techniques with relevance to glymphatic structure, flow, and function. We conclude by highlighting current barriers and future directions for translational imaging and applications to neurologic disorders.

## Brain compartments

The contents of the cranial vault include neurons (nerve cells), glia (supporting cells), vessels (arteries, capillaries, veins), and interstitium (everything else). Neurons are comprised of dendrites (reception of signals), cell bodies (processing of signals), and axons (transmission of signals across synapses). In the CNS, glial cells include astrocytes (ion homeostasis, blood flow, response to injury), oligodendrocytes (myelination), microglia (immune response), and ependymal cells (cerebrospinal fluid homeostasis). In the peripheral nervous system, the key players are satellite glial cells (surrounding cell bodies), myelinating and non-myelinating Schwann cells (surrounding axons), enteric glial cells (gastrointestinal tract), and olfactory ensheathing cells (olfactory system).^2^

Intracranial fluid distribution by volume includes CSF in the ventricles and subarachnoid spaces [10%], plasma within the vascular system [10%], intracellular fluid (ICF) within brain cells [68%], and interstitial or extracellular fluid (ISF, ECF) between cells and vessels [12%].^3^

## Neurofluid circulation and exchange

The Monro-Kellie doctrine holds that within the cranial vault, there is a reciprocal balance between the volumes of blood, brain, and CSF. However, the relative volumes and time scales of each component are different and vary dynamically with normal physiology as well as disease conditions.^4^ Recent experiments indicate that the arteriovenous system is a closed-loop multiscale vascular network, which is globally regulated and coupled to multicompartmental CSF dynamics.^5,6^ Current evidence suggests that neurofluid circulation and exchange between capillaries, ISF, and CSF occur globally throughout the neuraxis. Multiple patterns of neurofluid flow are driven by dynamic hydrostatic and osmotic gradients, which produce complex temporospatial changes in volume and pressure.^7–10^

### Cerebrospinal fluid physiology

Cerebrospinal fluid (CSF) functions include protection, nourishment, and waste removal throughout the neuraxis. CSF has a tightly regulated composition, consisting of 99% water along with various ions and macromolecules. Adult CSF volume is estimated at 150 ml, with 25 ml within the ventricles and 125 ml within the subarachnoid spaces of the brain and spine. Production is by choroid plexus within the ventricular system and ependymal cells lining the subarachnoid space. Secretion varies from 400 to 600 ml per day in healthy individuals, signifying a complete turnover of 4–5 times per 24 h.^3,11^

CSF demonstrates oscillatory motion on multiple time scales related to cardiac pulsations (0.8 s),^12^ pressure wave fluctuations (1 s),^13^ and respiration (3–4 s).^14^ Over a longer time scale (30 min), CSF demonstrates streaming bulk motion through the neuraxis, guided by the balance between secretion and absorption, and aided by ciliated ependyma.^15,16^ Over time, there is net CSF movement from the paired lateral ventricles via the foramina of Monro into the midline third ventricle. Focal narrowing at the aqueduct of Sylvius produces regional oscillatory flow, the overall direction of which is determined by age and disease process.^17,18^ Distal to the cerebral aqueduct lies the fourth ventricle, at which point CSF can pass inferiorly through the obex of the fourth ventricle into the central canal of spinal cord. Additional outflow pathways include the midline foramen of Magendie and lateral foramina of Luschka, opening to the cisterna magna and infratentorial space. Within the cranial vault, CSF can ascend through the supratentorial subarachnoid space toward the superior sagittal sinus. CSF also flows down through the foramen magnum to surround the spinal cord and nerves.^19^ Most importantly, CSF flows around cerebral perforating vessels along leptomeningeal sheaths and basement membranes (paravascular and perivascular spaces).^20^

### Cerebrospinal fluid efflux

CSF can exit the central nervous system via multiple pathways, ultimately draining to either the lymphatic or venous systems. Perineural drainage to peripheral lymphatics can occur through exiting cranial and spinal neurovascular sheaths, particularly the olfactory nerve fibers penetrating the cribriform plate.^21^ Meningeal lymphatics run along cerebral arteries and veins and connect to the glymphatic system. Multiple networks are present, including parasagittal dural spaces that drain to dorsal dural lymphatics and arachnoid granulations, and basal dural lymphatics that drain directly to cervical lymph nodes.^22^ Spinal lymphatics drain the dura mater in metameric circuits that connect to lymph nodes and the thoracic duct. Arachnoid granulations (villi) represent protrusions of the arachnoid mater through the dura mater into dural venous sinuses, and are the only known pathway for CSF drainage directly into the bloodstream.^23^

### Glymphatic system

Outside the neuraxis, peripheral lymphatics communicate with leaky capillary beds to maintain homeostasis by delivering nutrients and removing waste products. Within the neuraxis, however, capillaries are surrounded by tight junctions that restrict molecular transport and insulate the neural microenvironment. These endothelial barriers allow for transmembrane passage of water, ions, and small molecules, but exclude larger entities such as cells, protein, and glucose.^24^ Thus, for many years, it was a mystery as to how brain homeostasis, neurofluid exchange, and molecular communication with other organ systems was achieved.

**Figure 1.**
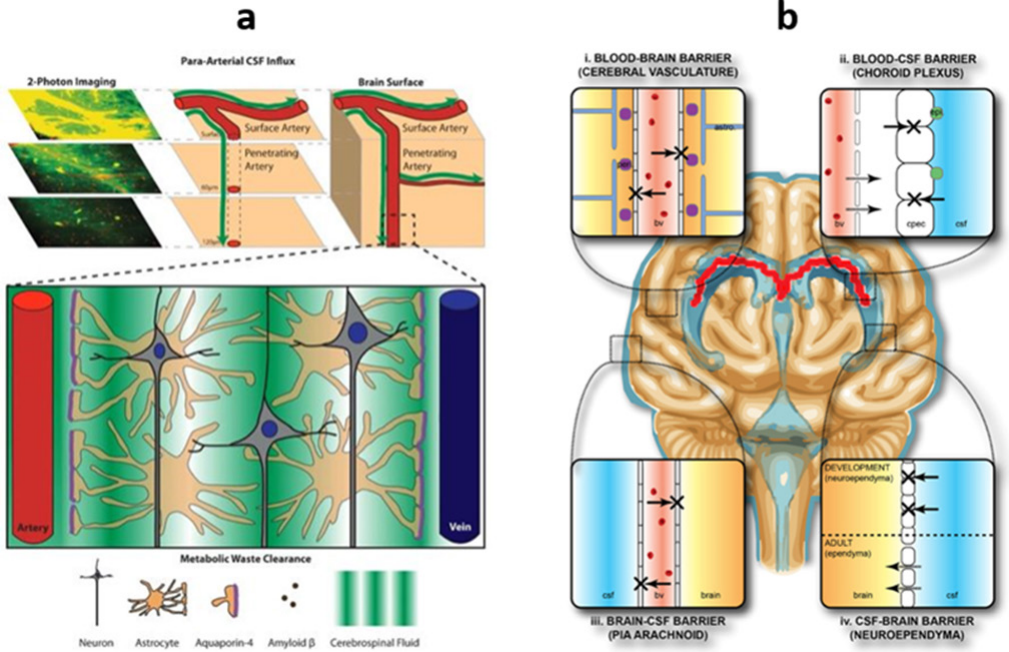
Anatomy of neurofluid exchange. (**a**) Glymphatic system. CSF flows into arterial paravascular spaces, undergoes capillary-level exchange with ISF modulated by AQP4 channels on astrocytic endfeet, and exits via venous paravascular spaces. Adapted from Wikimedia Commons, author Jeffrey J. Iliff, MeSH D000077502. (**b**) Brain barrier systems. (i) The blood–brain barrier is formed by endothelial tight junctions, pericytes in the basement membrane, and astrocyte endfoot processes ensheathing capillaries. (ii) The blood–CSF barrier consists of fenestrated capillaries lining the choroid plexus. (iii) The outer CSF–brain barrier represents the leptomeninges (arachnoid and pia mater), which are covered by endothelial intercellular junctions. (iv) The inner CSF–brain barrier is comprised of ventricular ependymal cells.^26^ Courtesy of Wikimedia Commons, CC BY-SA 3.0. CSF, cerebrospinal fluid; ISF, interstitial fluid.

In 2012, a group of researchers at the University of Rochester headed by Maiken Nedergaard used two-photon microscopy with fluorescent tracers to visualize CSF flow from the subarachnoid space into and through brain parenchyma in living mice. This resulted in the discovery of what they termed the “glymphatic” system, reflecting the key role of glial cells with a function analogous to conventional lymphatics. In their experiments, CSF was seen entering arterial paravascular spaces, combining with ISF and parenchymal solutes at the level of the capillary bed, and exiting venous paravascular spaces. They determined that the majority of CSF-ISF exchange is modulated by aquaporin-4 (AQP4) water channels on astrocytic endfoot processes that form glial limiting membranes around vessel walls^25^ (Figure 1a). Subsequent studies have identified separate perivascular spaces formed by leptomeningeal reflections from the surface of the brain, with additional clearance into the extracellular space along basement membrane layers within vessel walls. Overall CSF flow is multidirectional and dispersive, representing a combination of advection (bulk flow driven by arterial pulsations) and diffusion (concentration-dependent flow down gradients). Together, these processes allow for transport and exchange of both small and large molecules into the CSF.^27^

### Neurogliovascular unit

The neurogliovascular unit (NGVU) is the basic structural and functional unit of the brain. It is comprised of cells and extracellular matrix components that govern the normal cerebral hemodynamic response, regulating the blood supply to neural tissues. In healthy subjects, neurovascular coupling of neural activity and cerebral blood flow requires complex multidimensional signaling and coordination. In various neurologic diseases, NGVU dysfunction can result in neurovascular uncoupling and cerebral blood flow dysregulation. Recent studies suggest that NGVU function is largely regulated by glial cells including astrocytes, pericytes, and myocytes.^28–30^

## Barrier systems

Blood–organ barriers in the body enable selective molecular exchange and immune sequestration between the blood and other systems. Examples include the blood–gas barrier for pulmonary air exchange, blood–testis/blood–follicle barriers for germ cell development, and blood–thymus/blood–marrow barriers for immune cell development. These molecular “barriers” are actually semi-permeable membranes consisting of endothelial, epithelial, and/or mesothelial cells. Depending on molecular size and polarity, there are various mechanisms for transport through cell membranes (transcellular) as well as between cells (paracellular).

Within the neuraxis, several molecular barriers exist to regulate the neural environment. These barrier systems mature during fetal and postnatal development, and help to protect the CNS from infectious and toxic exposures, though they also present challenges for therapeutic delivery. In the brain, there are four major barrier systems: the blood–brain barrier (vessels), blood–CSF barrier (choroid plexus), outer CSF–brain barrier (leptomeninges), and inner brain–CSF barrier (ependyma)^31^ (Figure 1b).

### Blood–brain barrier

The blood–brain barrier (BBB) consists of endothelial tight junctions, pericytes embedded in basement membranes, and astrocyte endfoot processes ensheathing capillaries. This system restricts solute exchange between the circulating blood and ISF, allowing passive diffusion of water, gases, and small non-polar molecules, but also active transport of metabolites (ions, glucose, amino acids) critical for neural function. The BBB restricts passage of circulating toxins and pathogens that can cause brain parenchymal damage.^31^

Certain specialized structures in the brain lack a BBB, instead consisting of highly permeable fenestrated capillaries and specialized ependymal cells. These structures are known as circumventricular organs (CVOs) and modulate rapid communication between the CNS and peripheral targets. CVOs are located in the “AV3V” area, near midline around the anteroventral third and fourth ventricles, which are exposed to the highest flow rates of CSF and blood. They serve sensory (area postrema, subfornical organ, vascular organ of lamina terminalis) and/or secretory (subcommissural organ, pituitary, median eminence of hypothalamus, pineal gland) functions.^32^

### Blood–cerebrospinal fluid barrier

The blood–CSF barrier (BCSFB) is comprised of fenestrated capillaries lining the choroid plexus (CP). CP is highly vascularized, lacks the endothelial tight junctions present in the rest of the brain, and is convoluted with epithelial cells having aquaporin-1 (AQP1) barriers that rapidly secrete CSF in the absence of an osmotic gradient. Therefore, the BCSFB is the most permeable of the barriers, representing the major site for active transport and/or synthesis of large macromolecules, and a key target for intracranial drug delivery.^33^

### Brain–cerebrospinal fluid barriers

The outer CSF–brain barrier is comprised of the leptomeninges, namely arachnoid and pia mater. These contain specialized intercellular junctions (tight and adherens) junctions linking endothelial cells over the surface of the brain, which help to limit parenchymal invasion by immune and tumor cells. The leptomeninges are also closely associated with glymphatic and dural lymphatic drainage pathways.^34^

The inner brain–CSF barrier is lined by the ventricular ependyma, a single layer of ciliated epithelium that secretes, circulates, and maintains homeostasis of CSF. Ependymal cells provide cellular guidance during brain development, scavenge and detoxify contaminants, and transport some electrolytes and solutes.^35^

### Extracranial barriers

Additional blood–neural barriers are associated with the extracranial CNS and peripheral nervous system (PNS). Though less well-studied than in the brain, these systems demonstrate analogous formation of neurogliovascular units that ensure neural homeostasis and maintain barrier integrity. Specialized barrier systems within the globe, inner ear, spine, and peripheral nerves are critical for maintenance of proper neurologic function. Alteration of these barriers is closely related to development of neurologic disorders and affects the success of treatment approaches.^36^

### Blood–ocular barriers

Blood–ocular barriers are molecular barriers between blood vessels and various parts of the globe. In the healthy eye, these systems are essential for normal visual function with maintenance of the aqueous and vitreous humor. These barriers help to restrict hematogenous spread of infection and toxins, but can also impair local drug delivery. The blood–aqueous barrier is formed by epithelial cells of the ciliary body and endothelial cells of iris capillaries. The inner blood–retinal barrier represents the retinal vascular endothelium, which surrounds retinal capillaries with tight junctions similar to those in the brain. The outer blood–retinal barrier is the retinal pigment epithelium, with tight junctions that prevent passage of large molecules from underlying choriocapillaries.^37^

### Blood–labyrinth barrier

The blood–labyrinth barrier of the inner ear is responsible for maintaining the ionic microenvironment needed for mechanotransduction by auditory hair cells. This function is performed by the stria vascularis, a highly vascular tissue that produces and maintains endolymphatic fluid in the scala media of cochlea. The stria vascularis is demarcated by tight junctions between marginal cells at the medial secretory border and basal cells at the lateral cochlear border. Between these two layers, a perivascular intrastrial space contains strial capillaries, pericytes, and intermediate cells surrounding capillaries.^38^

### Blood–spinal cord barrier

The blood–spinal cord barrier (BSCB) is comprised of endothelial tight junctions, pericytes in basement membranes, and astrocytic endfeet surrounding capillaries. Though similar in structure to the BBB, the BSCB is overall more permeable, making it more vulnerable to disruption and disease processes, as well as a useful target for CNS drug delivery.^39^

### Blood–nerve barrier

The blood–nerve barrier (BNB) communicates with the BSCB along the dorsal root ganglia, which are lined with AQP1 channels that modulate pain reception via the dorsal spinal horns. The BNB regulates the microenvironment of peripheral nerve axons and Schwann cells, which are more penetrable than the BSCB. It consists of the endoneurial microvessels within nerve fascicles and their investing perineurial sheaths, composed of epithelial barrier membranes that are sealed by tight junctions.^40^

## Physiologic variation

Glymphatic function is modulated by various physiologic processes including the cardiac cycle, respiration, neural activity, and regional vasodynamics. In healthy volunteers, there are demonstrable effects on neurofluid flow when changing respiratory rate and mode, cardiac flow and pulsation, and cranial and spinal position.^41–43^ Further studies have reported alterations in glymphatic function related to other factors such as normal development and aging, lifestyle and diet, body position and exercise, and sleep and anesthesia states. In Nedergaard’s original study, glymphatic system activity nearly doubled during sleep, with up to 60% volume expansion of the extracellular space.^27,44–47^

## Disease pathology

Glymphatic system failure appears to represent the final common pathway for a variety of acute and chronic neurologic disorders. Inciting factors can include sleep disturbances; mechanical trauma; edema; hemorrhage; metabolic disruptions; inflammatory conditions; and microstructural abnormalities of membranes, barriers, or vessels. Over time, these conditions can yield irreversible impairment of neurofluid dynamics, termed “glymphedema” or “neurofluidopathy.”^48^ Ongoing accumulation of waste proteins (such as tau, β-amyloid, α-synuclein) further impairs glymphatic clearance and tissue function, resulting in progressive neural dysfunction and degeneration.^49^

In the brain, glymphatic dysfunction has been demonstrated in neurodevelopmental/neurodegenerative disorders; stroke/vascular disease; trauma; hydrocephalus/CSF pressure disorders; tumors; epilepsy; meningitis; demyelinating diseases; drug/toxin exposures; metabolic encephalopathy; headache; and neuropsychiatric conditions including sleep, pain, and mood disorders. Within the orbit, disordered fluid exchange is theorized to result in edema, inflammation, and vision loss. For the inner ear, disruption of fluid balance is seen in normal aging (presbycusis), infection (labyrinthitis), endolymphatic hydrops (Menière disease), and other causes of ototoxicity. The spinal cord and nerves show impaired fluid dynamics in neurodegeneration, ischemia/vascular lesions, trauma/compressive myelopathy, syrinx, tumor, infectious/autoimmune disease, and peripheral neuropathies.^48–51^

## Interventional therapies

As discussed above, the glymphatic system is critical for preserving neural health, and gradually fails in various neurological disorders. Therefore, better understanding and optimization of glymphatic function may enable more robust disease monitoring and early interventions to modify disease course.^27,52^ Successful administration of CNS therapeutics also relies on traversal of the glymphatic system, using either direct or indirect approaches. For example, CNS drug delivery can be achieved via direct injection into the CSF (intraventricular, intracisternal, intrathecal). These approaches physically bypass the BBB, but are invasive and carry risks of neurovascular injury and infection. Another option is to temporarily disrupt the BBB, either chemically or mechanically using focused ultrasound. Nanoparticulate systems that are small and lipophilic (liposomes, lipid nanoparticles, polymeric nanoparticles, micelles) can be administered peripherally, since they are designed to interact with the BBB at a molecular level. In the future, strategies to modulate glymphatic function and brain barriers could help optimize the success of neurointerventional procedures (catheterization, implantation, stimulation, ablation, surgery) in conjunction with other adjuvant therapies (immunotherapy, radiotherapy, chemotherapy) to maximize patient outcomes.^26,53^

## Diagnostic imaging

Experimental interrogation of neurofluid dynamics is complex and multifaceted. Past work in the field largely involved *ex vivo* and *in vitro* studies, with limited *in vivo* assessment of living animals or humans, resulting in many incorrect assumptions and faulty conclusions. Ongoing research is now being performed at multiple scales: molecular/cellular biology, tissue/organ evaluation, and patient/population-level studies. For human subjects, diagnostic imaging plays an important role in non-invasive or minimally invasive evaluation. However, there are several technical challenges involved in imaging the glymphatic system: multilevel interdependence of processes, need for both spatial and temporal resolution, and dynamic interplay between CSF and ISF.^27^

Multiple radiologic techniques have been applied to glymphatic imaging. The leading modality is MRI, which utilizes non-ionizing radiation and a wide variety of pulse sequences to investigate various tissue and flow properties. Modalities using ionizing radiation have been studied in animal systems: fluoroscopy or CT for cisternography, PET (positron emission tomography) for metabolism, and single photon emission computed tomography (SPECT) for flow. In human subjects, radiation-based approaches are impractical for research, but may be useful for clinical assessment of disease.^26^

MRI approaches to neurofluid evaluation can be classified by location (brain, spine), compartment (blood, CSF, ISF), use of contrast (non-contrast, gadolinium, ferumoxytol), timing (early, delayed) and technique (anatomic, diffusion, perfusion, etc.). Imaging techniques can be utilized to interrogate tissue properties (structure, flow, metabolism) during normal conditions, physiologic perturbations, and/or true disease states. The current literature includes imaging of both animal and human subjects for a variety of neurological disorders, including dementia, tumor, stroke, trauma, epilepsy, and demyelinating disease.^48–51^ Current barriers to clinical translation include incomplete biologic understanding, modality limitations as mentioned above, wide physiologic and technical variability, and lack of validation against clinical metrics.^54^

### Structure

**Figure 2.**
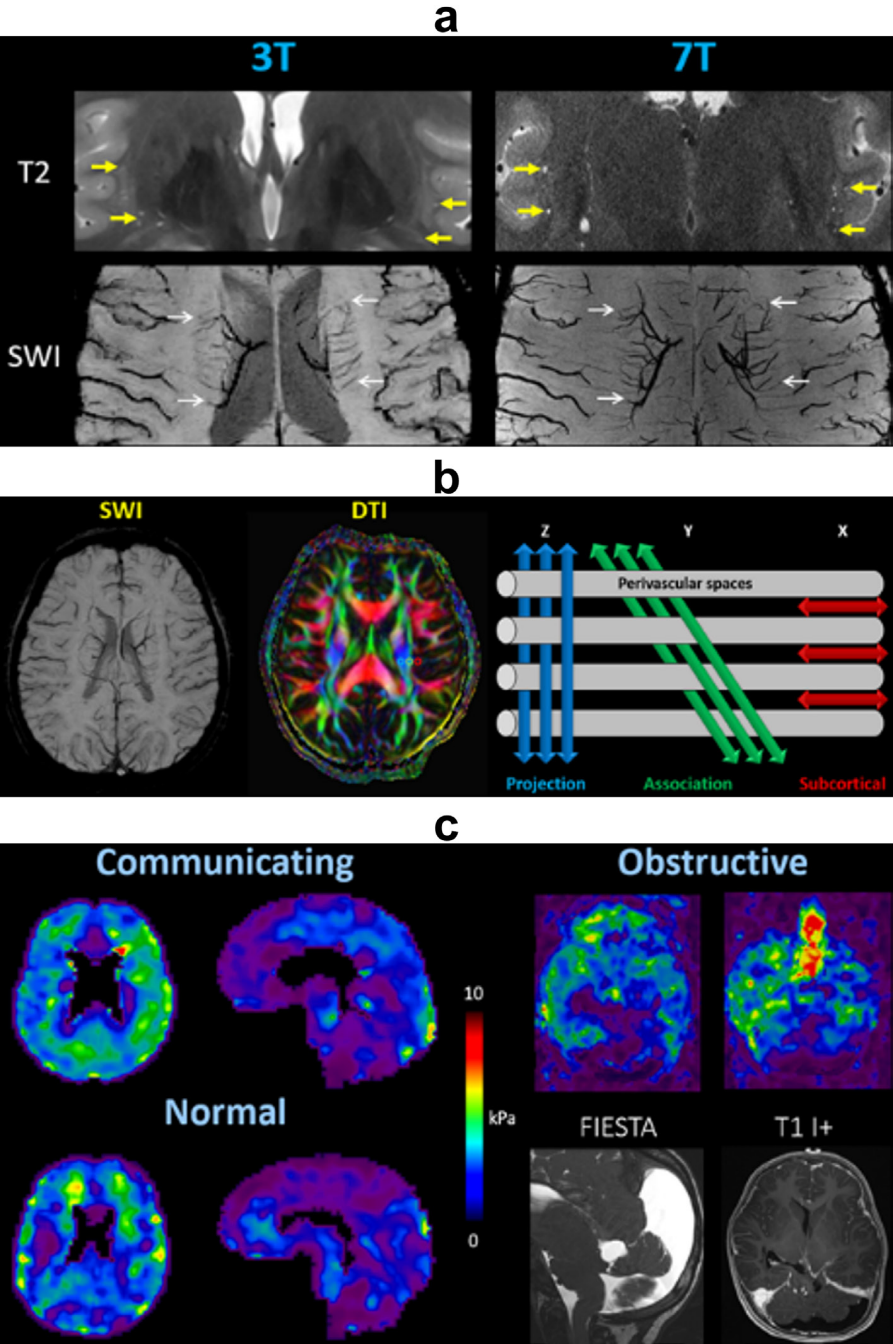
Structural imaging. (**a**) PVS are better delineated on ultra-high-field (7 Tesla) MRI compared to 3 Tesla, due to the higher SNR and spatial resolution. PVS appear as CSF-filled spaces (thick yellow arrows) with hyperintense signal on T2-weighted imaging, and surround penetrating small vessels (thin white arrows) with hypointense signal on SWI. (**b**) DTI-ALPS index. Blue: projection fibers of corticospinal tract in Z-axis (superior–inferior), green: association fibers of superior longitudinal fasciculus in Y-axis (anterior–posterior), red: subcortical fibers of subcortical U tracts in X-axis (left–right). The ALPS index is computed as the average diffusivity along the PVS (X-direction) divided by average diffusivity in perpendicular white matter tracts (Y- and Z-directions). (**c**) Magnetic resonance elastography shows stiffer brain parenchyma in communicating hydrocephalus and obstructive hydrocephalus compared to normal controls. ALPS, ALong the Perivascular Spaces; CSF, cerebrospinal fluid; DTI, diffusion tensor imaging; FIESTA, fast imaging employing steady-state acquisition; kPa, kilopascals; PVS, perivascular spaces; SWI, susceptibility-weighted imaging.

Perivascular space (PVS) imaging can be performed with standard T2-weighted MRI to identify CSF surrounding penetrating cerebral vessels. Artificial intelligence (machine learning and deep learning) algorithms have been developed to automatically segment PVS and calculate total volume, density, length, and tortuosity. Overall, PVS metrics increase with normal aging, disease processes, and loss of BBB integrity.^55^ Several approaches exist to visualize small cerebral arteries and veins using magnetic resonance angiography (MRA) or susceptibility-weighted imaging (SWI).^56^ These tiny vessels are optimally visualized at 7 Tesla or ultra-high-field (UHF) strength, which provides high signal-to-noise ratio (SNR) and spatial resolution (Figure 2a). Multiparametric techniques can be used to quantify and map white matter, gray matter, and CSF within the ventricles, subarachnoid space, parasagittal dura, leptomeninges, and perivascular spaces.^57^ Slightly different values within CSF compartments reflect the gradient of water concentrations among these structures, forming pathways for clearance of macromolecular waste materials.^58^

Diffusion-weighted imaging (DWI) and diffusion tensor imaging (DTI) can be used to measure the molecular diffusion of water using various gradient strengths or b-values. Brain diffusion demonstrates strong directional anisotropy along myelinated white matter tracts, as well as flow along perivascular spaces reflective of glymphatic activity. Taoka et al. have defined a readily calculable metric known as the ALPS (Along the Perivascular Spaces) index. On axial images at the level of the corona radiata and bodies of lateral ventricles, the medullary vessels and surrounding perivascular spaces extend out from the lateral ventricles in the X-direction (left–right). DTI fractional anisotropy (FA) color maps can be used to identify major white matter tracts from medial to lateral: blue projection fibers of the corticospinal tract (Z-axis: superior–inferior), green association fibers of the superior longitudinal fasciculus (Y-axis: anterior–posterior), and red subcortical fibers of the subcortical U tracts (X-axis: left–right). Glymphatic function can be estimated by the relative flow along PVS compared to the projection and association fibers. Therefore, the ALPS index is computed as the average diffusivity parallel to the PVS (X-direction) divided by average diffusivity in perpendicular white matter tracts (Y- and Z-directions) (Figure 2b):

$$ALPSindex=\frac{mean(D_{xproj},D_{xassoc})}{mean(D_{yproj},D_{zassoc})}$$

ALPS values are inversely correlated with normal aging^60^ and disease severity,^61^ processes that impair glymphatic flow along the perivascular spaces.

Magnetic resonance elastography (MRE), which utilizes external vibrations to estimate tissue stiffness, has been applied to diseases of the liver and more recently the brain.^62^ In animals and humans with hydrocephalus, increasing intracranial pressures correlate with increased brain tissue stiffness, indicating decreased compliance of both the brain parenchyma and CSF spaces (Figure 2c). These conditions presumably result in decreased glymphatic function, which may become directly measurable with improvements in spatial resolution and separation of directional components. Aging and disease studies have demonstrated correlations between increased arterial stiffness (both intracranial and peripheral) and greater perivascular space burden, decreased cerebrovascular reactivity, more brain parenchymal abnormalities, and poorer neurocognitive outcomes.^63^

### Flow

#### Non-contrast perfusion

**Figure 3.**
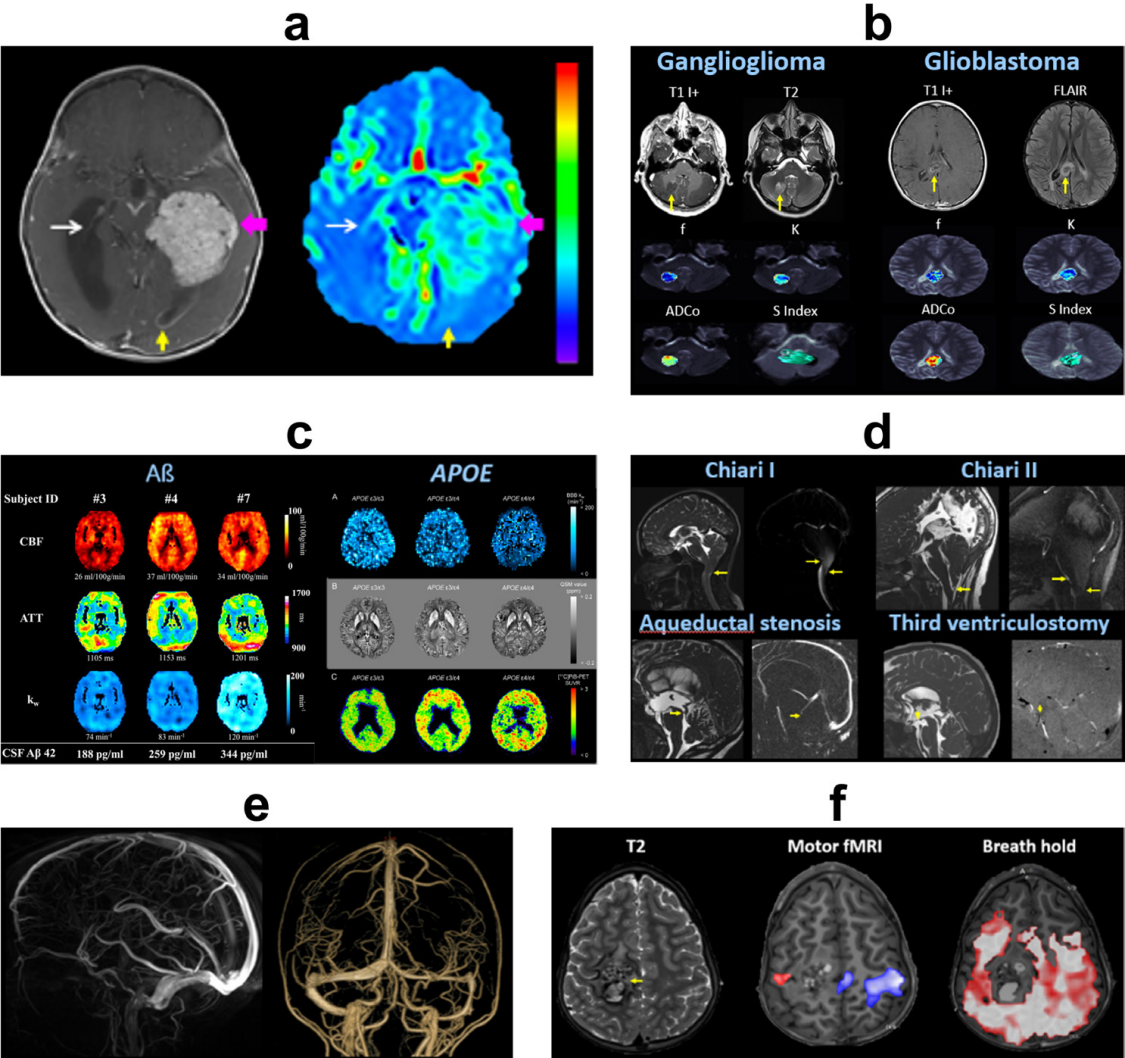
Non-contrast flow imaging. (**a**) ASL of atypical choroid plexus papilloma. Cerebral blood flow is markedly elevated in the tumor (pink arrows), moderately elevated in the left occipital horn due to regional leptomeningeal seeding (yellow arrows), and mildly elevated in normal choroid plexus (thin white arrows) compared to normal brain parenchyma. (**b**) IVIM of low- and high-grade gliomas. *f* = vascular volume fraction, *K* = kurtosis deviation, ADCo = corrected ADC, S Index = signature index. Courtesy of Denis Le Bihan, PhD. (**c**) Left panel: Diffusion-prepared ASL in cognitively normal subjects shows CBF, ATT, and BBB water exchange rate (k_w_) of three participants with different CSF concentrations of β-amyloid 42 (Aβ42), which if not properly cleared can form amyloid fibrils and plaques in the brain. Higher Aβ42 levels in CSF correlate with higher cerebral perfusion, BBB permeability, and neuropsychological function, suggesting better glymphatic clearance from the brain parenchyma. Right panel: Diffusion-prepared ASL k_w_ map, QSM, and ^11^C PiB-PET in normal APOE non-carrier (ε3/ε3), heterozygote (ε3/ε4), and homozygote (ε4/ε4). The APOE ɛ4 gene increases risk for Alzheimer dementia due to inefficient removal of amyloid plaques. Increased mutational load correlates with decreased BBB permeability, increased brain iron deposition and β-amyloid concentrations, and lower neuropsychological scores. (**d**) 2D PC MRI with superior–inferior encoding shows altered CSF flow dynamics in Chiari I, Chiari II, aqueductal stenosis, and post-endoscopic third ventriculostomy (arrows). (**e**) 3D PC MRA showing cerebral arteries and veins with high spatial resolution. (**f**) BOLD fMRI of right perirolandic cavernous malformation (arrow). Motor fMRI with bilateral hand clenching task demonstrates activity in the left (blue) greater than right (red) motor cortex, without detectable BOLD signal in the region of the lesion. Breath-hold task shows diffuse CVR throughout the brain, except in the region of the malformation. This indicates regional NVU resulting in a false-negative fMRI. Direct cortical stimulation at the time of surgery confirmed that motor function was present in the region of the malformation. ASL, arterial spin labeling; ATT, arterial transit time; BBB, blood–brain barrier; BOLD, blood oxygen level dependent; CBF, cerebral blood flow; CVR, cerebrovascular reactivity; fMRI, functional MRI; IVIM, intravoxel incoherent motion; MRA, magnetic resonance angiography; NVU, neurovascular uncoupling; PC, phase contrast; QSM, quantitative susceptibility mapping.

Arterial spin labeling (ASL) is a non-contrast technique that utilizes magnetically labeled arterial blood water as an endogenous tracer. Following an inversion pulse and specified post-label delay (PLD), labeled protons flow into the head and enable assessment of cerebral blood flow (CBF). Within the brain parenchyma, labeled blood water exchanges across the BBB into the ISF, and across the BCSFB into the ventricular CSF.^64^ On ASL, normal choroid plexus (CP) CBF is several times higher than in cortex, indicating water transport from the arterial blood to CSF. CP and gray matter perfusion appear to follow an inverse relationship in both healthy and diseased states, suggesting underlying homeostatic regulation and compensation (Figure 3a).^65^ Multidelay ASL (MDASL) with multiple PLDs can be used to quantify barrier water flux, based on the exchange times of labeled blood with tissue and CSF compartments. Animal models show decreased BBB permeability in aging and disease processes, and increased permeability following stroke and mannitol administration, correlating with standard clinical metrics. ASL permeability metrics are similar to those obtained by contrast-enhanced MRI, and may even be more sensitive in early stages of disease since water has a low molecular weight.^66^

Diffusion-weighted imaging using low b-values is sensitive to pseudo-diffusion signal from microcirculatory flow, which is high in blood and low in tissue. These approaches can be extended to assess glymphatic function and barrier permeability. For example, intravoxel incoherent motion (IVIM) utilizes a combination of low and high b-values to model both diffusion and perfusion contributions.^67^ IVIM can show altered parenchymal diffusion, microvascular perfusion, and perivascular fluid motion associated with various neurologic disorders^68,69^ (Figure 3b). Diffusion-prepared ASL can also be used to compute water exchange and barrier permeability in normal and disease processes^70,71^ (Figure 3c).

Phase-contrast (PC) imaging is commonly utilized with 2D or 3D directional velocity encoding (V_enc_) to measure rapid flow velocities of CSF and large vessels (Figure 3d–e). 4D PC MRI synchronized with cardiorespiratory pulsations has recently been developed to assess whole-body CSF and vascular spatiotemporal dynamics.^72^ PC applications to slower glymphatic flow are technically limited, but have been demonstrated experimentally with multi-V_enc_ and stimulated echo preparation. PC has also been combined with ASL to measure residual signals in the superior sagittal sinus and estimate global water extraction of the whole brain.^73^ A related technique, time-spatial labeling inversion pulse (Time-SLIP), applies a spatially selective inversion pulse to CSF or vessels and follows labeled fluid as it flows and exchanges along the neuraxis.^74^ Both 4D PC and Time-SLIP demonstrate spatiotemporal gradients in CSF flow within the choroid plexus, perivascular, subarachnoid, and parasagittal dural spaces corresponding to flow along glymphatic pathways.^75^

Blood oxygen level-dependent (BOLD) functional MRI (fMRI) leverages differential T2* signal between oxyhemoglobin and deoxyhemoglobin to detect cerebral blood flow. In a healthy NGVU, increased regional brain activity invokes the hemodynamic response with increased blood flow after a short (5–15 s) delay. To test the integrity of the hemodynamic response, cerebrovascular reactivity (CVR) mapping can be performed using a vasoactive challenge such as breath-holding, inhaled carbon dioxide, or intravenous acetazolamide to induce vasodilation.^76^ The intact NGVU will show neurovascular coupling with increased CBF, while in a diseased NGVU (astrocyte and/or pericyte dysfunction), the vessels are unable to dilate appropriately and the normal CBF response is absent or blunted with false-negative fMRI signal^77^ (Figure 3f).

Magnetic resonance encephalography (MREG) is an ultrafast BOLD technique in which whole-brain coverage is performed in 100 ms, with Fourier analysis used to separate the complex pulsations of the cerebral mantle. This approach has revealed three distinct physiological mechanisms affecting CSF pulsations. Cardiac pulsations occur at frequencies of ~1  Hz and are centered in periarterial regions with centrifugal spread. Respiratory pulsations occur at frequencies of ~0.3  Hz and are centered in perivenous regions with centripetal spread. The final groups of pulsations shows very low (0.001–0.023  Hz) and low (0.023–0.73 Hz) frequency propagating with unique spatiotemporal patterns, theorized to be related to glymphatic transport.^78^

^17^O is a heavy stable isotope of oxygen that can be produced exogenously using a nuclear reactor coolant. Subjects can then inhale ^17^O_2_, which is converted to isotopically labeled water in the body; or have H_2_^17^O saline solution administered intravenously. ^17^O decreases T2 relaxation and enhances T1ρ relaxation, with MRI and MRS used to quantify CBF and water exchange.^79^

#### Intravenous contrast

Gadolinium-based (Gad) contrast agents in MRI equilibrate between the intravascular space and ISF over time. This phenomenon occurs even in healthy barrier systems, with variable accumulation and clearance in the CSF and parenchymal compartments.^80^ Since Gad-based contrast agents shorten both longitudinal and transverse relaxation, either T1- or T2-weighted sequences can be employed to detect changes in blood, CSF, and ISF. T1-weighted sequences are faster due to short repetition times (TRs), but can suffer from extravascular and/or partial volume effects. Also, T1 signal shows a biphasic relationship with Gad concentration, and may scale unpredictably with observed MRI signal changes. T2-weighted sequences have longer TR and TE, resulting in lower time efficiency but more effective blood and tissue suppression. T2 signal scales monotonically with Gad concentration, though there is a plateau at higher concentrations. Inversion recovery (IR)-based sequences, such as fluid-attenuation inversion recovery (FLAIR) and black-blood MRI, can be used to suppress CSF/ISF and blood signals. These approaches generate stronger contrast for Gad-induced signal changes, but also introduce longer imaging times that can limit temporal resolution.^81^

**Figure 4.**
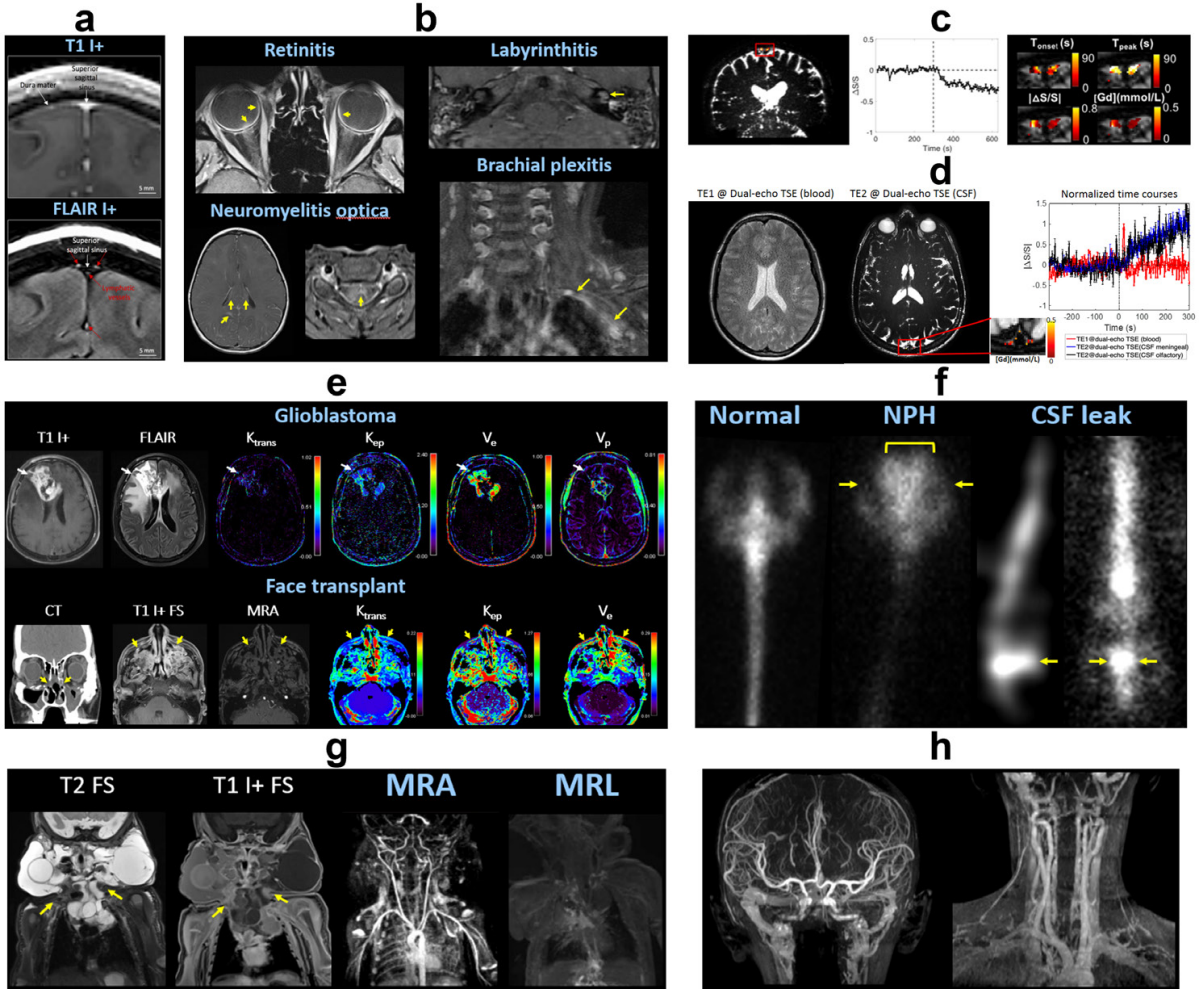
Contrast-enhanced imaging. (**a**) Meningeal lymphatics. Post-contrast T1-weighted MRI (top) shows enhancement of the dura mater and vascular structures (arrows). Post-contrast FLAIR (bottom) highlights the meningeal lymphatics (red arrows) in the parasagittal dural space, located between the cortical venous walls (white arrows) and pia mater. (**b**) Post-contrast T1-weighted MRI showing blood–neural barrier breakdown with abnormal enhancement in COVID-19 retinitis, left labyrinthitis, neuromyelitis optica, and left brachial plexitis (arrows). (**c**) DSC MRI using T2-weighted TSE shows dynamic signal changes in meningeal lymphatics (red box) surrounding the superior sagittal sinus. By plotting the signal over time relative to Gad injection (dotted line), several parameters can be extracted including T_onset_ = onset time, T_peak_ = time to peak, absolute value of relative signal change |ΔS/S|, and [Gd] = concentration of Gad. (**d**) Dual-echo TSE MRI can measure Gad-induced signal changes in blood at short TE (TE1) and CSF at longer TE (TE2), providing information about dynamic flow through blood vessels and meningeal lymphatics in a single scan. (**e**) DCE MRI of glioblastoma and facial transplant shows alterations (arrows) of various parameters including plasma volume V_p_, interstitial volume V_e_, transfer constants K_trans_ (from plasma to ISF) and K_ep_ (from ISF to plasma). (**f**) Radionuclide cisternography. Left panel: Healthy subject shows ascent of injected radiotracer through the lumbar spine to basal cisterns, cerebral convexities, and vertex. Central panel: Normal pressure hydrocephalus shows abnormal reflux into the lateral ventricles (bracket) and impaired ascent over the cerebral convexities (arrows). Right panel: Lumbar CSF leak with contrast pooling (arrows) and delayed ascent. (**g**) MRI, time-resolved MRA, and MRL of lymphatic malformation involving the neck and upper chest (arrows). There is Gad leakage within the malformation and disorganized drainage to the peripheral lymphatic system. (**h**) Ferumoxytol MRA. Arterial, capillary, and venous systems are concurrently opacified due to the long blood pool residence time. DCE, dynamic contrast-enhanced; DSC, dynamic susceptibility contrast; FLAIR, fluid-attenuated inversion recovery; MRL, MR lymphangiography; TE, echo time; TSE, turbo spin echo.

Qualitative evaluation of sequential Gad studies in normal subjects has been performed at short (5–30 min), intermediate (1–4 h), and long (4–24 h) delays after contrast administration. These studies show time-dependent transport of Gad from the choroid plexus into ventricular CSF, globe and inner ear, subarachnoid space, and neural parenchyma.^82^ Post-contrast FLAIR facilitates identification of meningeal lymphatics in the parasagittal dural space, located between the cortical venous wall and pial sheath (Figure 4a); as well as surrounding the middle meningeal artery and cribriform plate.^83^ Conditions that disrupt neural barrier integrity (aging, neurologic disease, exogenous agents) can accelerate Gad leakage. Therefore, rapid and abnormal enhancement along the neuraxis can be seen in various pathologic conditions including tumor, stroke, infection, inflammation, and trauma^84^ (Figure 4b).

Quantitative contrast-enhanced perfusion can be performed using dynamic susceptibility contrast (DSC) or dynamic contrast-enhanced (DCE) MRI.^85^ DSC uses T2*-weighted negative contrast, typically based off the first pass of Gad to calculate peak signal changes (Figure 4c). Using a dual-echo sequence, Gad-induced dynamic changes in both blood and lymphatic vessels can be measured in a single scan (Figure 4d).^86^ DCE applies extended T1-weighted positive contrast imaging to model effective volumes and partitioning between the intravascular space and ISF^87^ (Figure 4e). Temporal resolution for glymphatic imaging is lower than for standard blood perfusion, due to the requirement for whole-brain coverage with sufficient spatial resolution to image multiple fluid spaces.

#### Cerebrospinal fluid contrast

Direct injection of contrast into CSF is possible using a minimally invasive percutaneous approach. Access can be obtained at various locations along the neuraxis: higher levels decrease transit time to the cranial vault, but incur a greater risk of procedural complications. For clinical studies, Gad contrast can be safely used off-label at injection doses less than 1.0 mmol. Experimental animal and human studies have also utilized fluorescent, radioactive, or nanoparticle tracers.^88^ Myelography involves intrathecal (IT) access at the lumbosacral level, cisternography involves intracisternal (IC) injection at the craniocervical junction, and ventriculography involves intraventricular (IV) injection from a ventricular access device. After injection, the patient can be repositioned with a short time delay for contrast to disperse through the CSF and better outline the parenchyma (brain, spinal cord, nerves).^89^ Sequential T1-weighted MR myelography in normal subjects demonstrates Gad ascending up the spine to the basal cisterns and over the cerebral convexities, with perivascular dispersion and delayed parenchymal enhancement. Within the brain, Gad penetrates centripetally from the meningeal surface into the cortex and subsequently deep tissues, supporting the glymphatic hypothesis. The meningeal lymphatics are opacified last, indicating that they function downstream from the glymphatic pathway.^90,91^

Markedly abnormal CSF flow patterns are observed in normal pressure hydrocephalus, with reflux of injected contrast into the lateral ventricles (altered bulk flow) and impaired delivery to the cortex (decreased perivascular flow).^92^ Analogous patterns of normal and disrupted CSF flow in aging and disease have been observed in historical studies using fluoroscopic, CT, or radionuclide myelography/cisternography^93^ (Figure 4f).

A few centers have investigated delayed postcontrast imaging or transtympanic Gad injection for evaluation of Menière disease (endolymphatic hydrops). High-resolution MRI allows for distinct visualization of the contrast-enhanced perilymph from the enlarged endolymphatic sac within the membranous labyrinth.^94^

#### Lymphatic contrast

MR lymphangiography (MRL) can be performed non-invasively with injection of contrast material or radiotracers into regional veins, lymph nodes, or soft tissue; and invasively with selective access of lymphatic vessels.^95^ In the brain, dynamic imaging reveals drainage pathways via the meningeal and nasal lymphatic vessels into the deep and superficial cervical lymph nodes^96,97^ (Figure 4g). In the spine, lymphatic vessels form metameric circuits that connect the peripheral nerves and dorsal root ganglia, drain the epidural space and dura mater, and flow to connecting lymph nodes and the thoracic duct. Animal models of impaired lymphatic drainage show increased waste accumulation that correlate with aging and disease processes.^98^

#### Ferumoxytol contrast

Ferumoxytol is an ultrasmall iron oxide nanoparticle that is FDA-approved for treatment of iron deficiency anemia. It can be used off-label as an alternative MRI contrast agent, being generally safer and more biocompatible than Gad. The dosing is lower than for therapeutic indications (1 mg/kg), and administered as a slow infusion (over 1 h) to minimize the risk of anaphylactoid reactions. Ferumoxytol has strong T1 and T2 relaxation effects, with qualitative and quantitative imaging features similar to Gad. In normal subjects, there is immediate intravascular enhancement with a long blood pool phase (plasma half-life of 14 h) and progressive uptake by the mononuclear phagocyte system^99^ (Figure 4h). Meanwhile, there is slow leakage of ferumoxytol through an intact BBB. However, disorders that cause BBB breakdown and neuroinflammation (*e.g.* tumor, stroke, infection) result in increased ferumoxytol leakage and macrophage uptake with strong extravascular enhancement.^100,101^ Inherent biocatalytic activity and biodegradability of ferumoxytol also raise possibilities for combined MRI tracking and treatment delivery (theranostics).^102,103^

### Metabolism

Magnetic resonance spectroscopy (MRS) can resolve major proton metabolites including N-acetylaspartate (NAA) in neural tissue, creatine (Cr) from energy metabolism, choline (Cho) in cell membranes, lactate (Lac), lipids (Lip), and other macromolecules (MM). Metabolic alterations have been demonstrated for a variety of neurologic diseases in correlation with imaging features, disease severity, and clinical status.^104,105^

**Figure 5.**
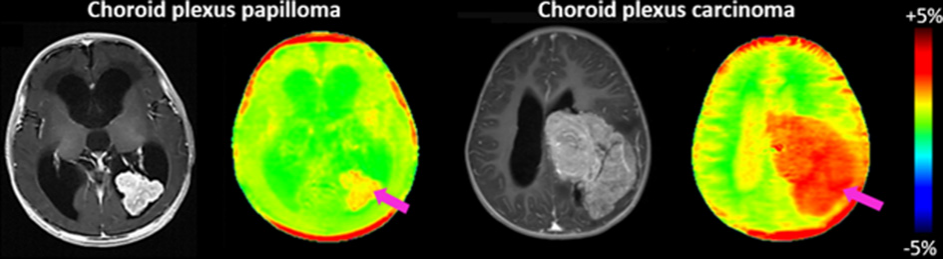
Metabolic imaging. APTw MRI of brain protein concentration. There is moderately increased APTw signal in choroid plexus papilloma and markedly increased signal in choroid plexus carcinoma (pink arrows) compared to the rest of the brain. Normal choroid plexus and CSF in the subarachnoid spaces contain low amounts of protein. Courtesy of Yun Peng, PhD. APTw, amide proton transfer weighted; CSF, cerebrospinal fluid.

Chemical exchange saturation transfer (CEST) detects small concentrations of exogenous or endogenous compounds, based on proton exchange with and subsequent reduction of free water signal, at a sensitivity over two orders of magnitude higher than MRS. Lymphatic,^106^ glucose,^107^ and amide^108^ CEST have demonstrated measurable signal alterations in animal models of neurologic disease (Figure 5).

Multinuclear (X-nuclear) imaging involves detection of physiologically relevant nuclei other than ^1^H, such as ^19^F, ^23^Na, ^35^Cl, ^37^Cl, ^39^K, ^17^O, and ^31^P.^109^ These techniques can yield additional information regarding ion transport (Na), metabolism (O, P, hyperpolarized ^13^C), and cell viability (Cl and K).^110^ X-nuclear MRI has important implications for comprehensive evaluation of metabolite distribution and clearance within the central nervous system.^111^

## Conclusions

The glymphatic system is responsible for maintaining homeostasis within the central nervous system, including nutrient delivery, waste clearance, and consistency of the ionic microenvironment. It is comprised of glial cells and barrier systems that modulate neurofluid production, circulation, and exchange. Various imaging techniques can be applied to interrogate glymphatic function, though clinical translation is hampered by technical factors, normal physiologic variation, and incomplete understanding of disease mechanisms. Continued research and collaboration among physicians and scientists will play a central role in advancing our understanding of and ability to modulate glymphatic function in neurologic disorders.
